# The Quik Fix study: a randomised controlled trial of brief interventions for young people with alcohol-related injuries and illnesses accessing emergency department and crisis support care

**DOI:** 10.1186/1471-227X-14-19

**Published:** 2014-08-08

**Authors:** Leanne Hides, David J Kavanagh, Mark Daglish, Susan Cotton, Jason P Connor, Jan J Barendregt, Ross McD Young, Davina Sanders, Angela White, Lance Mergard

**Affiliations:** 1 Center for Youth Substance Abuse Research (CYSAR), School for Psychology & Counselling, Institute of Health & Biomedical Innovation (IHBI), Queensland University of Technology (QUT), 60 Musk Ave, Brisbane, Queensland, 4059, Australia; 2 Center for Youth Substance Abuse Research (CYSAR), Faculty of Health Sciences, The University of Queensland, Brisbane, Australia; 3 Royal Brisbane and Women’s Hospital, Brisbane, Australia; 4 Centre for Youth Mental Health, University of Melbourne, Melbourne, Australia; 5 Orygen Youth Health Research Centre, Melbourne, Australia; 6 ChaplainWatch Inc, Brisbane, Australia

**Keywords:** Alcohol, Brief intervention, Young people, Substance use, Telephone, Motivation, Personality, Randomised controlled trial

## Abstract

**Background:**

Alcohol is a major preventable cause of injury, disability and death in young people. Large numbers of young people with alcohol-related injuries and medical conditions present to hospital emergency departments (EDs). Access to brief, efficacious, accessible and cost effective treatment is an international health priority within this age group. While there is growing evidence for the efficacy of brief motivational interviewing (MI) for reducing alcohol use in young people, there is significant scope to increase its impact, and determine if it is the most efficacious and cost effective type of brief intervention available. The efficacy of personality-targeted interventions (PIs) for alcohol misuse delivered individually to young people is yet to be determined or compared to MI, despite growing evidence for school-based PIs. This study protocol describes a randomized controlled trial comparing the efficacy and cost-effectiveness of telephone-delivered MI, PI and an Assessment Feedback/Information (AF/I) only control for reducing alcohol use and related harm in young people.

**Methods/design:**

Participants will be 390 young people aged 16 to 25 years presenting to a crisis support service or ED with alcohol-related injuries and illnesses (including severe alcohol intoxication). This single blinded superiority trial randomized young people to (i) 2 sessions of MI; (ii) 2 sessions of a new PI or (iii) a 1 session AF/I only control. Participants are reassessed at 1, 3, 6 and 12 months on the primary outcomes of alcohol use and related problems and secondary outcomes of mental health symptoms, functioning, severity of problematic alcohol use, alcohol injuries, alcohol-related knowledge, coping self-efficacy to resist using alcohol, and cost effectiveness.

**Discussion:**

This study will identify the most efficacious and cost-effective telephone-delivered brief intervention for reducing alcohol misuse and related problems in young people presenting to crisis support services or EDs. We expect efficacy will be greatest for PI, followed by MI, and then AF/I at 1, 3, 6 and 12 months on the primary and secondary outcome variables. Telephone-delivered brief interventions could provide a youth-friendly, accessible, efficacious, cost-effective and easily disseminated treatment for addressing the significant public health issue of alcohol misuse and related harm in young people.

**Trial registration:**

This trial is registered with the Australian and New Zealand Clinical Trials Registry ACTRN12613000108718.

## Background

Harmful or hazardous alcohol use is endemic among young people in the developed world [1-3]. In Australia, 60% of young people over a 12-month period reported drinking at levels placing them at risk of harm, with up to 30% drinking at levels placing them at weekly or greater risk of injury [1]. Hazardous alcohol use is a major preventable cause of injury, disability and death in young people, as well as a range of adverse mental health, social, educational, vocational and legal outcomes [4]. It is estimated to contribute to 27.5% of all deaths and 19.5% of substance-attributable disability-adjusted life years among 15–29-year-olds in the developed world [4]. In 2009, the economic burden of alcohol was estimated to equate to 0.45-5.44% of Gross Domestic Product (GDP) across 12 countries [5]. In Australia alcohol use represents 1.2% of GDP costing the economy $15 billion annually [6].

The rate of alcohol-related emergency department (ED) presentations in young people has increased dramatically in recent decades [7-9]. This presents a unique opportunity to engage traditionally non-treatment seeking youth into brief alcohol treatment. However, EDs are not ideal settings for the delivery of psychological treatments. Medical treatment for the injury or illness must take priority, and patients’ attention may be impaired by intoxication, distress or the administration of analgesics. An opportunistic intervention involving initial engagement in these settings, followed by delivery of a post discharge telephone-based intervention benefits both acute management and longer term treatment engagement. Global mobile devices and connections grew to 7 billion in 2013 [10]. Ninety-nine percent of young Australians own mobile phones, and overwhelmingly prefer electronic sources of health information over traditional ones [11,12]. Mobile phones provide an innovative, youth friendly and accessible way of delivering treatment.

Brief interventions (BIs) have a well established evidence-base for reducing alcohol use and related harm in adults [13,14], including adults presenting to EDs with alcohol-related injuries [15]. They typically comprise 1-2 sessions (from 10-15 minutes to 2 × 1 hour sessions) of personalised assessment feedback and information (AF/I). Many BIs include motivational interviewing, a person-centred therapeutic approach designed to assist individuals resolve ambivalence about problem drinking, set tangible goals for and strengthen commitment to change [16].

There is strong evidence for the impact of brief motivational interviewing interventions (MI) on young college students’ alcohol use and related problems at 3 [17-19], 6, 12, 24 and 48 months follow up, compared with no treatment [20,21]. One study has reported positive effects for MI on alcohol use and related problems among adolescents presenting to an ED with alcohol-related injuries at 3 and 6 month follow up, compared to standard care brief advice; [22].

While MI produces demonstrable reductions in alcohol use and related harm, there is significant scope to increase their impact. Most studies comparing MI and no treatment control conditions report small to moderate differences in alcohol use outcomes [20,21]. One reason for this is that treatment effects tend to reduce over time, as young people in control groups also begin to address their alcohol misuse [21,23].

It is unclear which form of BI is most effective for treating youth alcohol misuse as many studies report small effect sizes when comparing MI with other types of BIs. For example, while Monti *et al.*[24] reported ED-based MI was more effective for reducing alcohol use at 6 and 12 months follow up than assessment feedback alone, only small differences in effect size (f’s ranging -.24 to -.33) were found. We recently found two sessions of MI had more impact on alcohol use than an AF/I control at 1 (f = -.62) and 3 months (f = -.51) but not 6 month (f = -.47) follow up, in young people accessing a youth primary care service [25]. However, two further studies comparing MI with AF/I in college students at 6 and 12 month follow up, did not find superiority for MI [26,27].

Recent theoretical and empirical advances provide new insights into how to increase the impact of BIs for youth alcohol misuse. Most addiction theories focus on two major sources of reinforcement for substance misuse: (i) positive reinforcement linked to the pleasurable effects of substance use and (ii) negative reinforcement, linked to the relief of negative affect or withdrawal symptoms [28,29]. Personality risk models of substance use provide one way of understanding individual differences in susceptibility to drug reinforcement. Different personality traits have been shown to be reinforcement-specific, and are related to different patterns of substance misuse [30], distinct motivations for substance use [28,29,31] and differential sensitivity to alcohol reinforcement [32,33]. Four personality risk factors for youth substance misuse namely: (i) anxiety-sensitivity/proneness; (ii) depression-proneness; (iii) impulsivity-reward dependence; and (iv) sensation seeking, have been shown to differentially predict susceptibility to binge drinking, alcohol-related problems, illicit drug use and coping and enhancement motives for substance use in young people [30]. Anxiety proneness is a fear of anxiety-related bodily symptoms and has been linked to alcohol misuse and related problems in young adulthood, via a negative reinforcement process where alcohol is used to reduce anxiety symptoms and negative affect [34-37]. Depression-proneness links with drinking to cope with depression-specific emotions and is associated with early onset alcohol and drug problems [30,38]. Impulsivity or a tendency to act without thinking is associated with early onset and problematic alcohol and other drug use, as well as a range of other externalizing problems including antisocial and sexual risk-taking behaviors [38-44]. Sensation seeking or the need for intense pleasurable experiences, has the strongest links with alcohol misuse (especially binge drinking) and other drug use [45-47], and drinking for enhancement reasons via positive reinforcement processes [28,29,31].

Increasing evidence for a personality risk model of alcohol misuse has resulted in the development of brief personality-targeted interventions (PI), which differentially target the motivational pathways to alcohol misuse underlying these four personality dimensions. Conrod et al. [30] developed and evaluated the first brief PI incorporating psychoeducation and motivational and cognitive behavioral coping skills training among female adult substance users. This 90-minute BI was more effective for reducing alcohol misuse at 6 months, than a time-matched motivational film/supportive discussion or a mismatched intervention targeting another personality profile. Similarly, brief (3 x 1-hour sessions) group cognitive behaviour therapy targeting anxiety sensitivity in female students reduced hazardous alcohol use, conformity and emotional relief alcohol expectations at 10 weeks follow up, compared to control group seminar on psychology ethics [48].

Conrod et al. [49] developed a school-based group version (2 × 90 minute sessions) of PI, providing cognitive behavioral coping skills training targeting the motivational processes linking the adolescents’ dominant personality style to alcohol use. Three separate randomized controlled trials (RCTs) have demonstrated the efficacy of this approach for reducing the rates of alcohol use, binge drinking and alcohol-related problems, compared to no treatment at 4[49], 6[46,50], 12 and 24 month follow up [38,51]. This intervention also reduced growth in the quantity/frequency of alcohol use over time periods of up to 24 months, lessened the uptake of illicit drug use and reduced alcohol enhancement and coping motives at 24 months [38,51]. However, research is yet to determine the efficacy of PIs in young people with alcohol and/or illicit drug misuse, or compare its efficacy to other active BIs for alcohol misuse.

## Objectives of the study

We will conduct the first RCT to compare the efficacy and cost-effectiveness of three telephone-delivered brief interventions for alcohol misuse in young people. Participants will receive: (i) 1 session of assessment feedback and information (AF/I); (ii) 2 sessions of AF/I plus MI, or (iii) 2 sessions of a new personality-targeted intervention (PI) incorporating AF/I and MI. This study will identify the most efficacious and cost-effective telephone-delivered BI for reducing alcohol misuse and related problems in young people presenting to EDs/crisis support services. Telephone-delivered BIs provide an innovative, youth friendly and accessible way of delivering treatment to the 99% of young people who own a mobile phone.

*It is hypothesised that e*fficacy will be greatest for PI, followed by MI, and then AF/I at 1, 3, 6 and 12 months in terms of:

(i) the primary outcome variables of alcohol use and related problems, and

(ii) secondary outcome variables of mental health symptoms, functioning, severity of problematic alcohol use, alcohol injuries, alcohol-related knowledge, coping self-efficacy to resist using alcohol, and cost effectiveness

## Methods/design

### Trial design

**Figure 1:**
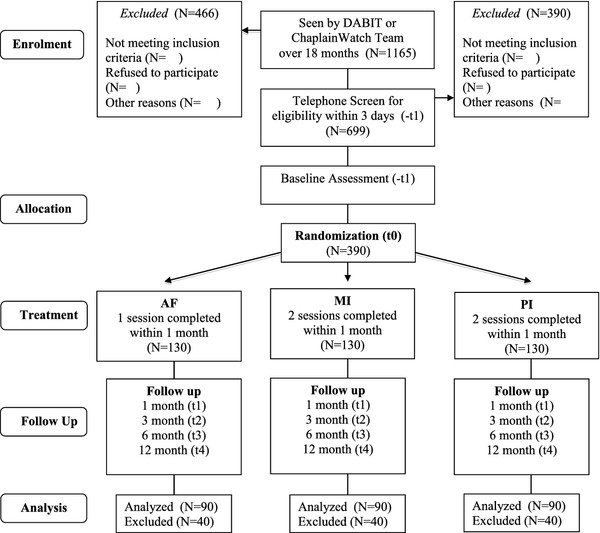
Projected CONSORT diagram, showing expected retention and loss.

This is a Phase II single blind superiority RCT comparing the efficacy of three BIs for young people presenting to a ED/crisis support service with alcohol-related injuries and illnesses: (i) 2 sessions of MI; (ii) 2 sessions of PI or a (iii) 1 session AF/I control. The design approximates an additive one, since MI includes AF/I, and PI incorporates AF/I, MI and personality specific coping skills training. Figure 1 provides an overview of the trial participant flow (See Figure 1). Participants are reviewed at baseline and 1, 3, 6 and 12 month follow-up.

### Study settings

Young people are recruited from two sites in Brisbane, Queensland Australia. The Drug and Alcohol Brief Intervention Team (DABIT) operates in the ED at Royal Brisbane and Women’s Hospital (RBWH), the largest hospital in Queensland. All young people referred to DABIT during office hours receive standard care comprised of 5-10 minutes of screening (daily alcohol, frequency of ≥ 6 drinks/occasion, use of illicit drugs/injected drugs), information and referral (e.g. withdrawal management) where appropriate.

ChaplainWatch (http://www.chaplainwatch.com) is an independent charity focused on public safety in public spaces. NightWatch Chaplains proactively patrol (via vehicle and foot) the inner-city entertainment precincts every Friday and Saturday night (as well as Special Events), from 11pm until 4-5am. They provide support, crisis intervention and conflict negotiation as well as frontline first-aid to any person in need, at risk or in crisis. A NightSafe Rest and Recovery Space is available for intoxicated, injured or sick persons. A registered nurse assesses and monitors vital signs and level of consciousness using the Glasgow Coma Scale (GCS), which provides an accurate assessment of consciousness in patients with acute alcohol intoxication [52]. Any patients with a GCS score of 8 or below or a suspected concussion/head trauma are immediately transferred to a local ED via ambulance.

### Eligibility criteria

Inclusion criteria are: aged 16-25 years, and either consumed ≥ 6 standard drinks on one occasion in the previous 2 weeks or scored ≥ 8 on the 10-item *Alcohol Use Disorders Identification Test* (AUDIT) [53]. Users of illicit drugs including cannabis are eligible, as long as alcohol is the most frequently used drug (other than tobacco). Exclusion criteria are: (i) ED serious medical problem or traumatic injury; (ii) not fluent in spoken or written English; (iii) unmodified hearing impairment; (iv) estimated IQ < 70 measured by the National Adult Reading Test; (v) current high suicide risk (current suicidal ideation/intent and plan); (vi) current or past history of psychosis; (vii) history of traumatic brain injury or organic brain disease (viii) currently in acute alcohol or drug withdrawal, and (ix) received psychological or pharmacological treatment for a drug or alcohol problem in the previous month.

### Sample size calculations

Sample size calculations were generated using SamplePower 3.0. Small to medium effects were anticipated between the three groups (effect size f = .25). This was based on Hides *et al*’s [25] finding of medium-to large effects at 1 (f = -.62), 3 (f = -.51) and 6 months (f = -.47) follow-up between MI and AF/I among young substance users in primary care and Monti *et al’s*[24] finding of small to moderate effects between MI and AF/I at 6 (f = -.24) and 12 (f = -.33) months follow-up in young alcohol users recruited from an ED. With power set at 0.95 and α to .05, 82 cases per group, or a total sample of 246 is required. We predict a 20-30% attrition rate based on our previous work and will therefore recruit a total of 390 participants over 18 months. With 82 young people in each group, we will have 88% power to detect small to moderate effects for the planned three group comparisons. Nevertheless, all young people will be included in the intent-to-treat analysis.

### Recruitment and follow up procedures

Approval to conduct the study has been obtained from the RBWH Human Research Ethics Committee (HREC; 13-23) and the QUT HREC (1300000391). Young people with alcohol-related injuries and illnesses identified by ChaplainWatch, DABIT or a research officer working across the two study sites, are asked if they are interested in participating in a research study on BIs for alcohol use, following completion of standard care. Those who express interest are provided with brief information about the study and their verbal consent and contact details (first name, mobile phone number and email address) obtained. De-identified information on the age, gender, and type of alcohol related injury/illnesses of all young people seen by ChaplainWatch/DABIT during project recruitment will allow us to compare the profile of our sample against that population.

Trained assessors with at least an Honours degree in Psychology contact the young person within 3 days, to provide further information on the study, obtain their informed consent and conduct a telephone eligibility screen. A 20-minute telephone baseline assessment is conducted either at this time or an appointment is made for a time more convenient to the young person. Participants are also required to complete online versions of self-report measures. Reporting on eligibility and refusal follows CONSORT Guidelines (Figure 1) [54].

Blind follow-up assessments consisting of a telephone interview and online survey are conducted by the research officer at 1, 3, 6 and 12 months. Follow-up appointments are made by phone, email and/or SMS 2 weeks beforehand, with a reminder at 1 week and 1 day before the appointment. The online survey is sent 1 week prior to the telephone interview. Online surveys not completed prior to the telephone interview are completed during the telephone interview. All participants are followed up, regardless of treatment completion. Assessors receive fortnightly supervision to monitor assessment, retention and blinding. Participants are reimbursed for their time related expenses on a rising reimbursement schedule where they receive $40 at baseline and 1 month follow up, $50 at 3 months follow up and $60 at 6 and 12 months follow up (total $250) to maximize retention. All baseline and follow up assessments are audiorecorded, and reliability and protocol adherence is assessed by an independent researcher on a random sample of 20% of participants.

### Randomization

An independent statistician generated a computerised random number sequence stratified by gender and age (16-20 years, 21-25 years), incorporating permuted blocks (blocks of 3 and 6) to allocate participants to one of three treatment groups (i) MI; (ii) PI or (iii) AF/I (Figure 1) [55]. The randomization sequence is concealed within a secure database, only accessible to the trial manager, who logs in to allocate participants when a baseline assessment is completed. The chief investigators, follow-up assessors and trial statistician are all blind to treatment group allocation.

## Psychological interventions

Research therapists with at least a Masters degree in Clinical Psychology attended a 1-day training workshop on AF/I, MI and the PI interventions lead by the first author using an adapted version of the Quik Fix MI Training Facilitator Manual [56]. Participants are randomized to receive (i) 2x 30min telephone sessions of MI; (ii) 2x 30min telephone sessions of PI or (iii) a 1×30 min telephone session of AF/I; (Figure 1). The same research clinical psychologist delivers both sessions of MI/PI over a maximum of 3 weeks to allow for missed appointments. Homework tasks are cued by emailed session summaries and brief SMSs.

All telephone treatment sessions are audio-recorded. Care is taken to ensure young people are in an appropriate setting during telephone consultations to ensure privacy and confidentiality. Group supervision meetings are held fortnightly to review a randomly selected session audio segment, independently rating it on the *Motivational Interviewing Treatment Integrity* (MITI 3.1.1) [57] and a *Session Component Checklist*, and discuss any departures from protocol. An independent psychologist, blind to treatment allocation, rates a random 20% sample of treatment sessions for treatment fidelity purposes.

(i) *AF/I:* The AF/I participants receive personalised assessment feedback on their alcohol use and information on the physical, psychological and social effects of alcohol use. AF/I is delivered sensitively, but MI principles are not used. The young person is emailed a copy of this information after the session and an SMS containing generic information on effects of alcohol use. Four subsequent SMSs are sent at 2, 4, 8 and 10 months post baseline containing this information.

(ii) *MI:* The MI comprises AF/I delivered in a MI style (i.e., empathically, amplifying the person’s own concerns). MI about alcohol is then provided to: (a) build readiness and commitment to make a change (including pros and cons of current use and change), (b) negotiate change goals, and (c) develop a plan for change. Individuals do not progress to Step b or c unless they are ready to change (i.e., rating ≥ 7/10 on *Importance of change & confidence to change* Likert scales). Homework (cued by emailed session summaries) for MI after Session 1 comprises review of their pros/cons balance sheet, and after Session 2, review of alcohol change plan or harm minimization strategies (if not ready to make a change). Brief post session SMSs remind the young person about the benefits of alcohol change they identified and any alcohol-related goals and plans. The researcher then makes four 5-10 minute phone calls to check-in with the young person and remind them of session content and conduct a *Timeline Followback* (TLFB) [58] for the past month at 2, 4, 8 and 10 months post-baseline.

(iii) *PI:* PI incorporates personality specific AF/I, MI and cognitive-behavioral coping skills training components. The intervention is designed to target how individuals with specific personality risk factors cope with their vulnerability to alcohol misuse. It is not intended to change personality, but to alter specific alcohol-related behaviors. In Session 1, the young person is given personalised AF/I, which not only includes a focus on alcohol use, but also includes psychoeducation about their dominant personality trait (anxiety proneness, depression proneness, impulsivity, or sensation seeking), associated problematic, personality-specific coping behaviors (e.g., avoidance, aggression, risky behaviors), and how this may affect their alcohol and other substance use. Using an MI style, therapists guide individual exploration of the personal relevance of these issues. Participants are given information on alcohol harm minimization strategies and are encouraged to think about how they could have used some of these strategies to prevent the recent alcohol-related injury or illness which resulted in their ED admission.

In contrast to standard MI, PI does not restrict discussion of potential change strategies to participants who elect to change their drinking. Instead, it encourages them to develop implementation intentions, a specific hypothetical or actual plan for change, which takes the form “if situation Y is encountered, I could cope more effectively by using Z, in order to achieve my alcohol use goal”, a technique based on the Theory of Planned Behavior [59]. A meta-analysis of 94 studies indicated implementation intentions were stronger predictors of behavioral action and change than generalised change plans [59]. Young people are guided to develop an implementation intention for future alcohol use situations. They are asked to identify an alcohol use situation in which they could test this alcohol plan in the next week and are asked to rate their level of confidence (1-10) in implementing the plan. As in MI, they recall past successes in behavior control to build their self-efficacy, and they identify and address any potential impediments. For homework, the young person is encouraged to implement their alcohol use plan, and is emailed a session summary and a brief SMS message as a reminder.

In Session 2, the young person’s success in implementing their alcohol use plan is reviewed and if necessary, the plan is revised. Young people then receive personality-specific training in two cognitive behavioral coping skills. Those predominantly with anxiety proneness are given training in mind chill thought awareness and acceptance training for anxious thoughts and mindful breathing. Young people high in depression proneness are provided with behavioral activation (looking after yourself/positive event scheduling) and thought awareness and acceptance training for depressive thoughts including refocusing their attention on more positive thoughts using the positive psychology three good things technique. Sensation seekers receive training in savouring techniques to increase their awareness and ability to focus on and experience positive feelings from everyday experiences (food, sex, music) and are encouraged to schedule natural highs from these as well as other functional activities (e.g. exercise, skateboarding, rock climbing). Young people with high levels of impulsivity receive mindfulness training to increase awareness of their impulsive urges and urge surfing techniques using the three D’s (delay, distract, decide). Those who experience difficulty identifying impulsive urges are encouraged to apply the Stop-Think-Do rubric to help them slow down their decision-making processes sufficiently to consider likely outcomes of behavioral alternatives. Session 2 concludes with the young person developing an implementation intention for future alcohol-related situations, which incorporates their personality-specific coping skills. They are encouraged to rehearse this plan in session, and identify and address potential impediments. A session summary and a SMS reminder of their alcohol use plan are subsequently sent. As in MI, the researcher then makes four 5-10 minute phone calls to check-in with the young person and remind them of session content and conduct a *TLFB* for the past month at 2, 4, 8 and 10 months post-baseline.

## Measures

Outcome measures are brief, to minimize assessment fatigue. The assessment schedule is provided in Table 1.

**Table 1:** Assessment schedule

**Measures**	**Timepoint**					
	**Recruitment**		**Follow ups**			
**(Timeframe)**	**Screen**	**Baseline**	**1 Mth**	**3 Mth**	**6 Mth**	**12 Mth**
	**(-t1)**	**(-t1)**	**(t1)**	**(t2)**	**(t3)**	**(t4)**
Demographics	X			X	X	X
Alcohol use disorders identification test (AUDIT)	X (3 mths)		X (1 mth)	X (3 mths)	X (3 mths)	X (3 mths)
Substance use risk profile scale (SURPS)		X			X	X
Timeline followback (TLFB; 1 mth)	X (2 wks)	X	X	X	X	X
Controlled drinking self-efficacy scale (CDSE; 3 mths)		X		X	X	X
Rutgers alcohol problem index (RAPI; 3 mths)		X	X (1 mth)	X	X	X
Alcohol trivia		X	X			X
University of Rhode Island Change Assessment Scale (URICA; current)		X	X	X	X	X
Extended-adolescent injury checklist (E-AIC; 6 mths)		X			X	X
Mental health: Kessler 10 (1 mth), Generalised Anxiety Disorder-7 (GAD-7; 2 wks), Mini Social Phobia Inventory (Mini-Spin; 1 wk); Patient Health Questionnaire-9 (PHQ-9; 2 wks)		X	X	X	X	X
Coping in stressful situations (CISS)		X		X	X	X
Social & role functioning (1 mth)		X	X	X	X	X
Client satisfaction questionnaire (CSQ-8)			X			
Other treatment service use			X	X	X	X
Assessment of quality of life-6D (AQoL-6D)		X		X	X	X

Note. mth: month; wk: week.

### Screening measures

(i) Demographics: age, gender, occupation, years of education, living arrangements, relationship status, ethnicity.

(ii) Alcohol use: A 2-week *TLFB* uses calendar-based cues to obtain precise information on the frequency and quantity of alcohol use [58]. The 10-item *AUDIT* screens participants for harmful or problematic drinking in the previous 12 months. This well validated measure reliably detects alcohol use disorders in adolescents and adults at a cut-off of ≥ 8 [53].

### Baseline and follow-up measures

(i) Personality risk: The *Substance Use Risk Profile Scale* (SURPS) [60] is a 23-item measure of the level of anxiety sensitivity/proneness (5-items), depression proneness/hopelessness (7-items), impulsivity (5-items), and sensation seeking (6-items) personality risk factors for alcohol abuse or dependence. This scale has high levels of internal consistency, test-retest reliability and construct, concurrent and predictive validity with respect to current and future alcohol use in adolescents, undergraduate students and clinical samples [30,60,61]. It also has high levels of incremental validity (cf. other personality measures) for differentiating reinforcement-specific patterns of drug use [29,60].

(ii) Alcohol and other drug use: *TLFBs* over the previous 4 weeks assess the frequency and quantity of alcohol and other substance use across the study [58]. The *TLFB* method has high test-retest reliability (intraclass correlation coefficients (ICC) = .70-.94), convergent and discriminant validity with other substance use measures, collateral information and biological measures of illicit drug use [58,62]. We found substantial agreement between urine drug screens and self-reported drug use (83%) using the TLFB in a previous study [63]. The age of onset and frequency of alcohol and other drugs use over the previous 12 months is also assessed at baseline.

(iii) Other alcohol outcome measures. Alcohol-related knowledge is assessed using 20 *Alcohol Trivia* questions developed for this study. Alcohol-related problems (eg., social, medical, legal, family, vocational) are assessed on the *Rutgers Alcohol Problem Index* (RAPI) [64], a 23-item self-report measure commonly used in BI studies. Alcohol-related injuries are assessed on the *Extended Adolescent Injury Checklist* (E-AIC) [65]. This 12-item measure records minor and major injuries, whether they required medical attention, and occurred in the context of alcohol or other drug use. The 12-item *University of Rhode Island Change Assessment Scale* (URICA) [66] is used to gauge motivation for changing alcohol use. The 15-item *Controlled Drinking Self-Efficacy Scale* (CDSE) [67], provides a reliable and valid measure of self-efficacy to resist excessive drinking.

(iv) Mental Health: Self-report information on the young person’s history of mental health and substance use disorders, as well as their family history is collected. The *Kessler 10* (K10) [68] is a 10-item self-report measure of psychological distress in the past month. Normative data indicates a cut-off of ≥ 17 is at the 75^th^ percentile among Australian youth [69]. Depression symptoms are measured using the 9-item *Patient Health Questionnaire-9* (PHQ-9) and anxiety symptoms are assessed by the 7-item *Generalised Anxiety Disorder-7* (GAD-7). Social phobia is measured by the 3-item *Mini Social Phobia Inventory* (Mini-SPIN).

(v) Coping and Functioning: Emotion and task-orientated coping are measured with 32-items from the *Coping Inventory for Stressful Situations* (CISS) [70] scale. The clinician-rated *Global Functioning: Social and Role*[71] scales provides two single ratings of the lowest level of social (e.g., peer, family) and role (school, work) functioning in the past month on a 1 (extreme dysfunction) to 10 (superior) scale. Both scales use detailed anchor points for each rating interval and probe questions to increase reliability.

(vi) Cost-Effectiveness: A total health care and personal cost perspective is taken; it does not include costs to society. Health care costs (government, insurers, patients) include the costs of treatment provision (staff costs, missed appointments, record keeping, training, materials, rent and overheads) and exclude research costs. As participants may seek other treatment elsewhere, information on all other health care service use is collected. Service prices are based on standard Australian Medicare and Prescription Benefit Scheme (PBS) schedules. The 20-item *Assessment of Quality of Life-6D* (AQoL-6D) for adolescents provides a measure of health-related quality of life on 6 dimensions (inc. social, mental health, independent living) [72]. It is the only multi-attribute instrument weighted with Australian preference values for use in economic evaluations.

### Treatment satisfaction and fidelity

Treatment satisfaction is assessed at one month follow up using the 8-item *Client Satisfaction Questionnaire-8* (CSQ-8) [73]. MI fidelity is confirmed on the *MITI 3.1.1*[57]. Session Component Checklists are also completed to ensure core features of the allocated treatment are delivered and non-allocated treatments are not delivered.

## Data, safety and risk management strategies

Safety and risk management protocols manage any safety or urgent treatment issues. Young people who are recruited to this study and are subsequently identified as in need of more intensive treatment will be appropriately referred. A clinical trials committee comprised of the authors meets every 3 months to monitor the project’s implementation (inc. study withdrawals), clinical (inc. any serious adverse events) and research (inc. data safety) integrity. Email or telephone consultations are used to determine urgent inclusion or treatment issues, and a record of decision precedents is kept to ensure consistency.

## Statistical analysis

The independent variable is treatment condition, with 3 levels: (i) AF/I; (ii) MI and (iii) PI. The primary outcome variables are alcohol use (TLFB) and related problems (RAPI). Secondary outcomes include mental health symptoms (psychological distress (K10), depression (PHQ-9), social anxiety (Mini Spin), generalised anxiety (GAD-7)), functioning (Social and Role Functioning scales), severity of problematic alcohol use (AUDIT), alcohol-related knowledge, alcohol-related injuries (E-AIC) and coping self-efficacy (CDSE) to resist using alcohol. Preliminary statistical analyses will be undertaken to check for baseline group differences on demographic, primary and secondary outcome variables using one-way analysis of variance (interval data) and chi-square (χ^2^) analyses (categorical data). Intention-to-treat strategies will be used for the main analyses. To determine whether there are group differences on the primary and secondary outcome measures at 1, 3, 6 and 12 months a series of mixed effects model repeated measures analyses of variance (MMRM) will be employed. The within groups factor will be time (baseline, 1, 3, 6 and 12 months) and group will serve as the between subjects factor. This technique can also control for potential confounding variables such as gender, age, injury severity and other drug use.

A two-pronged economic analysis will be conducted. An analysis alongside the trial will display the costs against their benefits in both natural units, such as alcohol-related injuries/illnesses avoided, and QALYs. Costs saved will also be estimated by quantifying visits to GPs and other health service use avoided under the intervention. MI and PI will be assessed against the AF/I arm as comparator. Uncertainty in the assessment will be estimated using non-parametric bootstrapping. In the second modelled CEA, the estimated change in alcohol consumption from the trial will be extrapolated to the general Australian population of 16-25 year olds. The interventions will be evaluated using a model developed for the Assessing Cost-Effectiveness of Prevention (ACE-Prevention) project [74], which determines DALYs averted by an intervention from changes in risk of 20 alcohol-related diseases and injuries. By using this model, we can compare cost-effectiveness of the PI and MI intervention with other alcohol interventions and up to 150 other preventive interventions (e.g., for illicit drugs, obesity, etc.) that have already been evaluated for the Australian population. Data on alcohol-related diseases and injuries from the Australian Burden of Disease 2003 study will be updated to 2015. Links between alcohol consumption and these diseases and injuries are based on the Comparative Risk Assessment study. The model is a multi-cohort, multi-disease model that follows up the target population until death or age 100. Uncertainty will be assessed using a combination of non-parametric bootstrapping for the effect size (alcohol consumption) and Monte Carlo simulation for all other variables (with sampling uncertainty).

## Discussion

Here we report the study protocol of an RCT comparing the efficacy and cost-effectiveness of three telephone-delivered BIs for young people with alcohol-related injuries and illnesses. The primary aim of the study is to identify the most efficacious and cost-effective telephone-delivered BI for reducing alcohol misuse and related problems in young people presenting to crisis support services/EDs.

There is strong evidence for the short-term efficacy of MI for reducing alcohol use in young people compared to no treatment. However, it is unclear if MI is the most efficacious and cost effective type of BI available as trials comparing MI with AF/I have given inconsistent results. Considerable debate remains about the most effective duration, content, context and mode of delivery of BIs for alcohol misuse, and there is significant scope to increase their impact. This study uses an additive design to address these research questions comparing (i) 1 session of AF/I which gives feedback and information, with (ii) 2 sessions of MI which includes AF/I, delivered in an MI style, plus other MI elements, to build readiness to change, negotiate change goals and develop a plan for change and (iii) 2 sessions of PI which delivers AF/I and MI incorporating personality-targeted cognitive behavioral coping skills training to determine if this increases MI’s impact. This will be the first study to examine the effectiveness of PI in young alcohol users outside of a group-based school prevention program and compare its efficacy to other BIs.

This design does have some limitations. It could be argued that the shorter duration of the AF/I control condition may be insufficient to produce an effect. However, the content of the AF/I condition can be easily delivered in one session and even briefer AF/I interventions have demonstrated efficacy in the clinical research literature. While the MI and PI interventions are time matched and delivered in two sessions to ensure an adequate dose of treatment is provided, it is possible that the overlaps in session content may wash out any differential treatment effects. However, there are significant and substantial differences between the BIs and treatment fidelity will be comprehensively assessed. Assessment reactivity as a result of the research process itself may potentially reduce any differential treatment effects. Reassuringly, only small assessment effects (<1 standard drink per week or a 1 point difference on the AUDIT) were found in a meta-analysis of 10 studies examining whether questions about alcohol use changed behavior among hazardous drinkers [75]. A finding recently replicated in university students receiving alcohol assessment and feedback, alcohol assessment only and a no contact control [76]. In the current study moderate effect sizes are expected, and any assessment effects occur across all conditions and are unlikely to affect comparative results. Additionally, all of our assessments are brief, and restricted to measures crucial to determine primary and secondary outcomes.

Engaging young alcohol users with alcohol-related injuries and illnesses who present to crisis support services and EDs into alcohol treatment is challenging, as they are unlikely to view their alcohol use as problematic or responsible for their injury or illness. Initial engagement in these settings, followed by rapid and assertive follow up and the provision of telephone-delivered BIs targeting their recent alcohol related injury or illness, provides a more youth friendly way of engaging young people into treatment than traditional services. Preliminary piloting of this process has produced positive results with 50-70% of young people referred to the study engaging in a BI depending on the referral source. The following strategies are also used to maximise participation and ensure dropout is below 30%: (i) obtaining multiple ways to contact participants at baseline; (ii) the development of tracking databases; (iii) adopting flexible appointment hours, giving pre-appointment reminders and rapidly following up missed appointments; (iv) a rising reimbursement schedule for the time associated with completing the baseline and follow up assessments, and (v) the assessment of only primary outcome measures when a young person wishes to discontinue treatment or is unwilling to undertake the full follow-up protocol.

The large numbers of young people presenting to crisis support services and hospital EDs with alcohol related injuries and medical conditions present a unique opportunity for engagement in BIs targeting their alcohol use. This may also reduce the risk of future alcohol-related injuries, illnesses, disability and mortality in young people, as well as a range of adverse mental health, social, educational, vocational and legal outcomes. The brief alcohol related interventions evaluated in this study can be easily transferred to existing health professionals across ED and other crisis services as well as primary care, mental health and alcohol and other drug treatment settings. Results of this project are expected to exert a significant influence on clinical policy and practice aimed at increasing access to evidence-based and cost-effective care for young alcohol users aimed at reducing the individual, community and economic costs of youth alcohol misuse.

## Abbreviations

AUDIT: Alcohol use disorders identification test; AF/I: Assessment feedback/information; AQoL-6D: Assessment of quality of life-6D; BI: Brief intervention; CDSE: Controlled drinking self-efficacy scale; CISS: Coping inventory for stressful situations; CSQ-8: Client satisfaction questionnaire-8; ED: Emergency department; DABIT: Drug and alcohol brief intervention team; E-AIC: Extended adolescent injury checklist; GAD-7: Generalised anxiety disorder-7; GDP: Gross domestic product; HREC: Human research ethics committee; K10: Kessler 10; MI: Motivational interviewing; Mini-SPIN: Mini social phobia inventory; Mth: Month; MITI: Motivational interviewing treatment integrity; MMRM: Mixed effects model repeated measures analyses of variance; PHQ-9: Patient health questionnaire-9; PI: Personality-targeted intervention; RAPI: Rutgers alcohol problem index; RCT: Randomized controlled trial; RBWH: Royal Brisbane and women’s hospital; SURPS: Substance use risk profile; TLFB: Timeline followback; URICA: University of Rhode Island change assessment scale; Wk: Week.

## Competing interests

LH developed the Quik Fix brief interventions which are the subject of this trial. These programs are distributed not for profit. The other authors declare that they have no competing interests.

## Authors' contributions

LH, DK, SC, MD, JC, AW, JB and RY are the Chief Investigators on the NHMRC grant in Australia. LH is responsible for ethics and clinical trial submission, recruitment, supervision of the baseline and follow up and delivery of the brief interventions. MD and LM are responsible for recruitment and data collection at DABIT and ChaplainWatch respectively. SC is responsible for data maintenance and analysis. All authors were involved in study design and will be involved in data analysis and reporting of the study results. All authors read and approved the final manuscript.

## Pre-publication history

The pre-publication history for this paper can be accessed here:

http://www.biomedcentral.com/1471-227X/14/19/prepub
